# Ethnotaxonomy of sharks from tropical waters of Brazil

**DOI:** 10.1186/s13002-018-0273-0

**Published:** 2018-11-21

**Authors:** Marcelo Moreira de Carvalho, Mônica Rocha de Oliveira, Priscila Fabiana Macedo Lopes, Jorge Eduardo Lins Oliveira

**Affiliations:** 1 Department of Oceanography and Limnology, Centre of Biosciences, Universidade Federal do Rio Grande do Norte—UFRN, Via Costeira Senador Dinarte Medeiros Mariz, Mãe Luíza, s/n, Natal, RN CEP 59014-002 Brazil; 2 Fishing Ecology, Management, and Economics Group, Department of Ecology, Centre of Biosciences, Universidade Federal do Rio Grande do Norte—UFRN, Campus Central- Avenue Senador Salgado Filho, Lagoa Nova, n°3000, Natal, RN CEP 59078-970 Brazil

**Keywords:** Bycatch fauna, Fishers’ local knowledge, Coastal fishing resources

## Abstract

**Background:**

Accessing folk knowledge from small-scale fishers is an affordable and reliable approach to understand the dynamic and diversity of shark species worldwide, especially of those eventually caught. In this context, ethnotaxonomy (folk identification and classification) may represent an alternative to support sharks fisheries management, especially in data-poor places. This study aimed to investigate fishing and ethnotaxonomy of the main shark species caught by small-scale fisheries from the coastal waters of the Brazilian Northeast.

**Methods:**

Semi-structured and structured interviews were conducted with fishers targeting general aspects of fishing activities and specific topics regarding ethnotaxonomy, capture, and commercialization of sharks. For species identification, an ethnobiological systematic perspective was used to analyze the folk nomenclature and classification criteria. Non-parametric statistical tests were used to verify associations between species caught, fishing gear, and harvest period.

**Results:**

Fishers mentioned 73 binomial names, 21 main folk species, and eight synonymies. Some species belonging to the same scientific genus are often named and grouped by the same folk name, with no distinction between species by fishers. Sharks are most landed as bycatch and correspond to less than 5% of the total commercial fisheries in the communities, with socioeconomic value for subsistence consumption and local commercialization. Sharks were said to be mainly caught with hand line and surface long line during the rainy season, while gillnet captures were associated to the dry season. At least three of the species most mentioned by fishers are currently classified as vulnerable and endangered worldwide.

**Conclusions:**

Even though landed sharks account for a small proportion of the fishing catches, their biological and life history features place sharks among the most vulnerable organisms globally. Such an ethnobiological approach towards shark identification may contribute to generate basic information on species caught, their frequency in the landings, and how different species belonging to the same genus can be landed and sold together. This type of information can generate subsidies to the development of conservation and management plans for these fishing resources, where knowledge is scarce.

**Electronic supplementary material:**

The online version of this article (10.1186/s13002-018-0273-0) contains supplementary material, which is available to authorized users.

## Background

Shark captures and the import of their meat have placed Brazil among the greatest consumers of these fishing resources, affecting species stocks of local and worldwide occurrence [1, 2]. This reality is even more concerning given that official statistical records, when existent, tend to register a substantial majority of the species landed under general categories such as *cações* (popular name for sharks, when commercialized), which compromises accurate monitoring and possible management of these fishing resources [3].

One of the main reasons for such difficulty to identify landed sharks is due to a high morphological similarity among different species [1, 3]. Furthermore, sharks were not commercially attractive until late 1980s, with little allocation of public funding to national programs for species identification, and fishing governance and enforcement [4]. However, over the last decades the demand for shark’s products (e.g., fins) to supply an expanding Asian market has boosted fisheries to unsustainable levels and practices, such as “finning”. The latter consists of removing the fins, with the animal usually still alive, and discarding the remaining animal’s body into the open sea, impacting stocks and generating an enormous amount of waste [2, 5].

Fisheries statistics for small-scale fisheries in Brazil was carried out by the government from 1990 to 2006, when it started being gradually discontinued, until it finally ended in most places by 2011 [6]. In the northeastern coast, the state of Rio Grande do Norte was among the main producers of sharks in 2006 (1311.5 t), where species were registered by their local names: *cação azul* (*Prionace glauca*), *cação lombo-preto* (*Carcharhinus falciformis*), *cação Panã* (*Sphyrna* spp*.*), *cação cavala* (*Isurus oxirinchus*), *cação tigre* (*Galeocerdo cuvier*), and just *cação* (any other shark) [7]. Some of these groups (e.g., *Sphyrna*) are currently undergoing population declines [2].

The biology of sharks, including their life history complexity, places these animals as important trophic regulators in marine ecosystems, but also puts them as one of the most vulnerable resources to overfishing [8]. For example, sharks in general exhibit low fecundity rate, long gestation periods (up to 12 months), and late sexual maturity, sometimes taking decades to copulate [9]. Excessive fishing has caused stocks of *Sphyrna lewini* and *Carcharhinus longimanus* to decline by more than 80% on the coast of Brazil [2]. In response, studies towards conservation and management of sharks have provided information on catches [2], political and social awareness [3], and species population structure [10].

Ethnoichthyological studies in fishing communities have contributed to access the local ecological knowledge (LEK) of fishers, clarifying important aspects related to their trophic ecology [11], behavior [12], and fishing and population declines [13]. Such wide array of information suggests the applicability of ethnoichthyology to obtain relevant information on shark ecology and support fishery management. Moreover, LEK approaches are strategic for consisting low-cost methods that make use of non-lethal technics to obtain data and for enabling access to unique life-experience information from the interviewees [13–15].

Ethnotaxonomy corresponds to a fundamental branch of ethnoichthyology used to understand the identification, nomenclature, and classification criteria of fish used by fishers [16–18]. In the ethnotaxonomical classification proposed by Berlin et al. [19], the living beings can be organized into six taxonomic levels: kingdom, life form, intermediate, generic, specific, and varietal. The “intermediate” and “varietal” levels are not common or are even inexistent in some cultures, explaining why it is more common to find ethnotaxonomical studies referring to the other levels, especially those more abundant in the natural world (namely, the “generic” and “specific” ones) [19].

Studies conducted with artisanal fishers along the Brazilian coast have demonstrated through fish nomenclature analyses and the types of relationships that different folk taxa represent to local communities by generic and specific terminologies [15, 20]. In addition, folk classification has shown its potential to support scientific research especially when data is limited and to contribute to fisheries decision-making [15, 20, 21]. Nevertheless, the use of ethnotaxonomy to understand shark uses and promote their management is still incipient globally and in Brazil, possibly due to their relatively lower participation on global catches scenarios compared to target fishing groups [22]. Despite the importance of sharks in an ecological and socioeconomic context, information concerning species composition in landings is still scarce, and when available, it is likely affected by misidentification. This is possibly the picture faced by sharks regularly landed in the Brazilian northeast, where not much is known about their capture and commercialization [23, 24]. The current study aimed to present ethnotaxonomy as an affordable approach to identify the main shark species captured and commercialized by small-scale fisheries from the state of Rio Grande do Norte, generating support for management and conservation measures for these fishing resources.

## Methods

### Study area

**Fig. 1:**
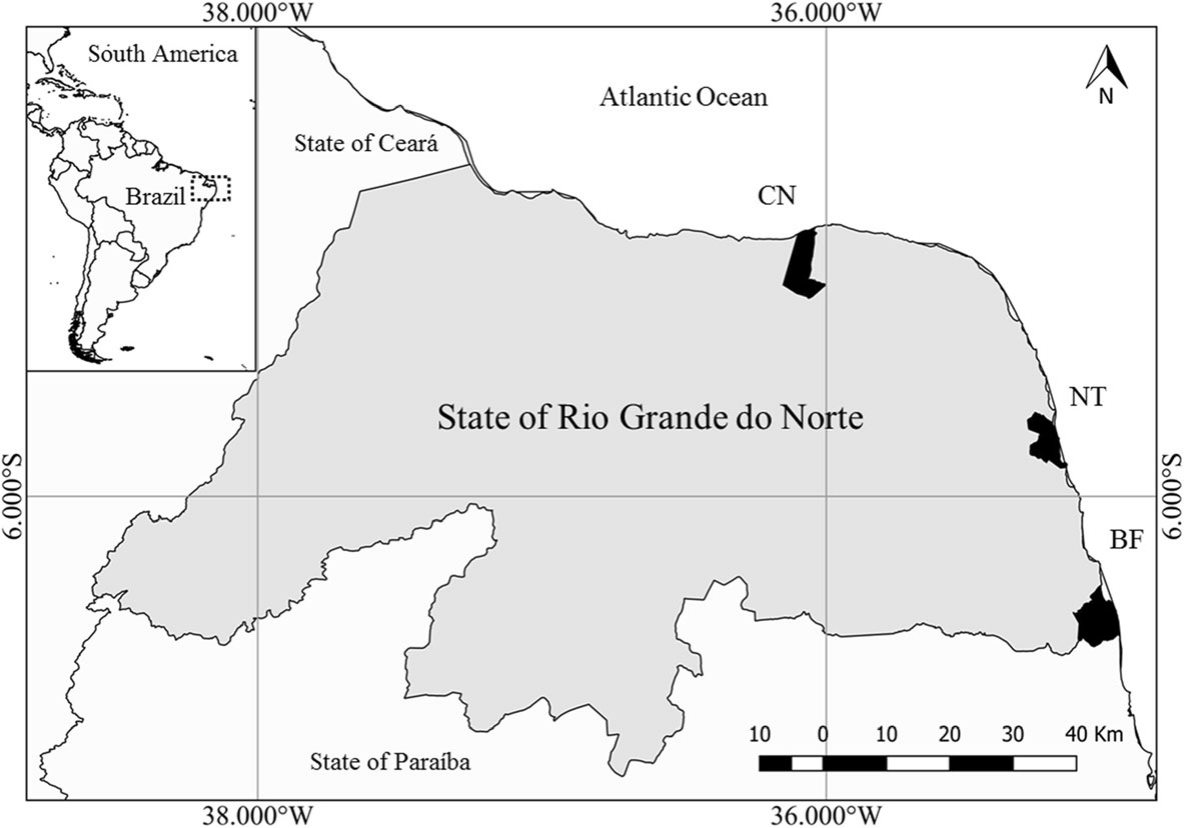
Geographical location of the state of Rio Grande do Norte, northeastern of Brazil, highlighting the sampling sites at the municipalities of Caiçara do Norte (CN), Natal (NT), and Baía Formosa (BF)

The state of Rio Grande do Norte (5.4026° S, 36.9541° W) (Fig. 1), northeastern of Brazil, extends for about 410 km along the Western Atlantic coast and presents a narrow continental shelf (63 km average width) that breaks at 60 m of depth [25]. Its tropical weather has a mean annual temperature of 26.5 °C, marked by a rainy season occurring from January to August [26]. The last detailed fishery data for municipalities indicates small-scale fisheries accounted for over 120 tons of landed sharks in 2006 [7], representing 0.75% of the landed fish by this sector.

### Methodology

Semi-structured and structured interviews were used to gather information from working and retired fishers in the state of Rio Grande do Norte, specifically from the northwestern town of Caiçara do Norte, the central-east city of Natal (capital of the state), and the southeastern town of Baía Formosa between December 2017 and February 2018 (for the Interview Form see Additional File 1). The respondents were those with a minimum fishing experience of 15 years in the region and who fished with small vessels (up to 15 m). Fishers were previously informed about our goals, joining in only those ones who consented written or orally.

The questions comprised general aspects of fishing activities in the region, aiming to verify the following: mean species compositions based on landed fish (target commercial species), fishing gear, type of vessels, fishing season (the most frequent period for shark incidental capture), distances traveled to reach fishing grounds, and average fishing time.

Twenty photographs of the most frequent shark species in the region [27, 28] were presented to fishers. Photographs were numbered, randomly organized and displayed on the same order to each participant [18, 29]. Among the species, *Rhizoprionodon porosus* was chosen as “positive control test” because it is a species regularly landed by small-scale fisheries in the Brazilian Northeast [23, 30], whereas *Negaprion brevirostris* was chosen as “negative control test”, because its distribution in the South Atlantic Ocean is closely restricted to oceanic islands, with inexpressive captures in inshore waters [31]. The use of control species intended to verify the reliability of shark species identification by fishers [20]. Fishers were asked to provide the local (folk) names of the sharks presented in each photograph and their main morphological criteria used for recognizing/identifying the species.

A subsample of 30 fishers was randomly selected (10 from each sampling site) to answer a structured form (binary format) related to folk classification of sharks based on the following morphological (size, shape and color) criteria: rounded/sharped head, short/long snout, small/large mouth, small/large eyes, large/narrow width fin, small/large length fin, small/large body size, light/dark color head, light/dark color fins, and light/dark color body (for details on the Specific Interview Form see Additional File 2). Fishers answered specific questions about shark fishing and commercialization of products and byproducts from local fisheries. In addition, small-scale fisheries landing records provided by the two sampled towns (Municipal Secretary of Fishing, Agriculture and Husbandry of Caiçara do Norte and Baía Formosa) for the years 2015 and 2016 were also accessed. These statistics were used to extract the representation of sharks in total landings from small-scale fisheries.

### Data analyses

The rarefaction test, as an estimator for sampling species richness [32], was used in this study to verify the richness of folk nomenclature. Each folk name given by the fishers was counted, tabulated, and compared to an estimated bootstrapped value (Jackknite 1 estimator) by using PAST 3.17 Software. Species folk nomenclature based on the identification from photographs was analyzed by ranking the most cited folk names and their respective relative percentage values on descending order. As proposed by Silvano et al. [21], we considered the main folk species those that were cited at least by 15% of the interviewees. Shark folk names were lexically analyzed for generic and specific terms (binomials), which correspond to terminal levels 2 and 3, respectively, considering the nominative criteria used by fishers [19].

For folk classification based on morphological criteria (size, shape and color), data were organized into a contingency matrix from the sum of binaries values (0/1) obtained by each criterion. Then, the matrix was analyzed by Unweighted Pair Group Method with Arithmetic mean (UPGMA), using Bray-Curtis similarity index on PAST software [18].

Categorical data related to general fishing aspects were analyzed by the non-parametrical tests Kruskal-Wallis and chi-square. Data related to shark local capture were analyzed by multiple correspondence analysis (MCA) on XLSTAT 19.7 software in order to verify any possible associations between fishing gear, fishing season, and main captured shark species. Information related to shark meat and byproducts commercialization was qualitatively analyzed and described. Prices for shark meat and byproducts were converted to US dollar considering purchasing power parities (PPP) for the year of 2017. It was considered a *p* value significance of 0.05 to all tests conducted in this study.

## Results

Due to the similar responses among sampling sites, all data were pooled into a single dataset for the state of Rio Grande do Norte. A total of 308 male fishers (Caiçara do Norte = 107; Natal = 104; Baía Formosa = 97), aging from 18 to 87 years old (mean 43.46 ± 12.79), who had been fishing in the region for about 26.21 ± 12.74 years, were interviewed. Small-scale fisheries in Rio Grande do Norte commonly involve low-cost fishing gears, such as handlines (46.34%), surface/bottom gillnets (23.67%), and longlines (12.05%). Other modalities (spearfishing, bottom traps, and trawl net) were also mentioned (17.94%). Fishing vessels are small-sized (e.g., sailboats, motorboats, rowboats, and canoes) with an average of 9 ± 2.8 m long, few exceeding 15-m size. These vessels operate within 27.30 ± 21.57 nautical miles from the coast. Fishing trips last mostly between 4 and 5 days, with occasional boats from Natal spending 15 days at sea.

### Ethnotaxonomy of the main shark species

The identification of the photographs of *R. porosus* (68%—more than 15% for each folk name) and *N. brevirostris* (24%—less than 15% for each folk name), used as control species, supports the reliability of the interviewees’ responses for the rest of the shark species used in this study. Fishers name, identify, and classify inshore shark species with relative detailing. Sharks local names were linguistically analyzed as secondary lexemes (binomials), generated by the polytypic generic noun *cação* (shark) plus an ethnospecific modifier term, which in general refers to morphological (animals and objects) and ecological (habitat and behavior) aspects related to those sharks.

For example, *Alopias superciliosus* was mainly identified as *cação raposa* (literally, fox shark), an association based on the similar morphology between the shark caudal fin and a fox tail. On the other hand, binomials generated for *Ginglymostoma cirratum* were based on both a morphological aspect (c*ação lixa—*sandpaper), referring to the species rough skin texture and to an ecological aspect (*papa-terra*—sand-eater), referring to the species feeding habits associated to benthic substrates (Table 1).

**Table 1:** Brazilian Portuguese nomenclature (generic name *cação* + ethnospecific modifier) for the main sharks species identified by fishers from small-scale fisheries from the state of Rio Grande do Norte, northeastern of Brazil

Species scientific identification	English common nomenclature	Brazilian Portuguese ethnospecific modifier	Identification per species (%)	Capture citation	IUCN/MMA status
*Alopias superciliosus* Lowe, 1841	Bigeye thresher shark	**RAPOSA** (*fox*)^1^	136 (67)	2	VU/VU
		RABUDO (*long-tailed*)^1^	17 (8)	–	
		GAIÚDO/GALHUDO (*long-tailed*)^1^	3 (1.5)	–	
		ZORRO (*caudal fin in ‘z’ shape*)^1^	1 (0.5)	–	
		misidentification	47 (23)	–	
*Isurus oxyrinchus* Rafinesque, 1810	Shortfin mako	**CAVALA** (*mackerel fish*)^1^	121 (49)	42	VU/ NT
		**BRANCO** (*white*)^1^	83 (33)	32	
		ANEQUIM (*agressive*)^2^	14 (6)	–	
		misidentification	30 (12)	–	
*Galeocerdo cuvier* (Péron & Lesueur, 1822)	Tiger shark	**JAGUARA-PINTADA** (*jaguar*)^1;2^	107 (48)	17	NT/NT
		**TIGRE** (*tiger*)^1;2^	74 (33)	2	
		misidentification	41 (18)	–	
*Prionace glauca* (Linnaeus, 1758)	Blue shark	**TOALHA** (*towel*)^1^	172 (60)	42	NT/NT
		**AZUL** (*blue*)^1^	73 (25)	20	
		misidentification	44 (15)	–	
*Rhizoprionodon lalandii* (Müller & Henle, 1839)	Brazilian sharpnose shark	**RABO-SECO** (*thin tail*)^1^	87 (43)	28	LC/NT
		**FRANGO** (*chiken*)^1^	31 (15)	–	
		misidentification	85 (42)	–	
*Rhizoprionodon porosus* (Poey, 1861)	Caribbean sharpnose shark	**RABO-SECO** (*thin tail*)^**1**^	90 (53)	35	LC/DD
		**FRANGO** (*chiken*)^1^	25 (15)	–	
		misidentification	56 (33)	–	
*Sphyrna lewini* (Griffith & Smith, 1834)	Scalloped hammerhead shark	**PANÃ** (*hat*)^1^	147 (51)	76	EN/CR
		PANÃ AMARELA (*yellow hat*)^1^	28 (10)	–	
		**MARTELO** (*hammer*)^1^	76 (26)	–	
		misidentification	39 (13)	–	
*Sphyrna mokarran* (Rüppell, 1837)	Great hammerhead shark	**PANÃ** (*hat*)^1^	156 (54)	74	EN/EN
		**PANÃ AMARELA** (*yellow hat*)^1^	43 (15)	–	
		**MARTELO** (*hammer*)^1^	83 (29)	–	
		misidentification	5 (2)	–	
*Ginglymostoma cirratum* (Bonnaterre, 1788)	Nurse shark	**LIXA** (*sandpaper*)^1^	243 (80)	11	DD/VU
		**PAPA-TERRA** (*sand-eater*)^2^	57 (19)	–	
		misidentification	4 (1)	–	
*Carcharhinus falciformis* (Müller & Henle, 1839)	Silky shark	LOMBO-PRETO (*black back*)^1^	17 (12)	6	NT/NT
		misidentification	121 (88)	–	
*Carcharhinus leucas* (Müller & Henle, 1839)	Bull shark	**CABEÇA-CHATA** (*flat head*)^1^	40 (26)	–	NT/NT
		misidentification	116 (74)	–	
*Carcharhinus limbatus* (Müller & Henle, 1839)	Blacktip shark	**SICURI-DA-GALHA-PRETA** (*rainforest snake with blacktip fin*)^1^	64 (36)	15	NT/NT
		SICURI (*rainforest snake*)^1^	18 (10)	–	
		misidentification	96 (54)	–	
*Carcharhinus perezi* (Poey, 1876)	Caribbean reef shark	**CABEÇA-DE-CESTO** (*basket head*)^1^	47 (26)	18	NT/VU
		CABEÇUDO (*bighead*)^1^	6 (3)	–	
		misidentification	126 (70)	–	
*Carcharhinus signatus* (Ranzani, 1839)	Night shark	**NOTURNO** (*Nightly*)^1^	43 (41)	–	VU/VU
		SICURI-BOLA (*ball shape snake*)^1^	14 (13)	–	
		misidentification	48 (46)	–	
*Carcharhinus acronotus* (Poey, 1860)	Blacknose shark	**FLAMENGO** ^**1**^	188 (65)	36	NT/NT
		misidentification	100 (35)	–	
*Carcharhinus plumbeus* (Nardo, 1827)	Sandbar shark	BICO DOCE-DE-PAREDE (*from the continental slope*)^1;2^	12 (10)	–	VU/CR
		misidentification	105 (90)	–	
*Negaprion brevirostris* (Poey, 1868)	Lemon shark	LIMÃO (*lemon*)^1^	12 (13)	–	NT/VU
		DOS-RECIFES (*from reefs*)^2^	10 (11)	–	
		misidentification	72 (77)	–	
*Sphyrna zygaena* (Linnaeus, 1758)	Smooth hammerhead shark	**PANÃ** (*hat*)^1^	175 (72)	22	VU/CR
		**MARTELO** (*hammer*)^1^	46 (19)	–	
		misidentification	23 (9)	–	
*Rhincodon typus* Smith, 1828	Whale shark	**PINTADINHO** (*flacked*)^1^	147 (63)	–	VU/VU
		**BALEIA** (*whale*)^1^	65 (28)	–	
		ESTRELA (*star*)^1^	12 (5)	–	
		misidentification	8 (3)	–	
*Carcharhinus obscurus* (Lesueur, 1818)	Dusky shark	FIDALGO (*noble*)^1^	11 (11)	–	VU/EN
		misidentification	85 (89)	–	

Ethnospecific terms based on the following: ^1^morphological criteria; ^2^ecological criteria. Names in bold correspond to the main folk species cited (minimum citation of 15%). Fisher’s capture based on the number of valid citations within the last 12 months before interviews (misidentifications were not considered). Conservation status according to the International Union for Conservation of Nature (IUCN) and Brazil’s Ministry of the Environment-(MMA) Ordinance 445 of 2014 [53]. *EN* endangered, *VU* vulnerable, *LC* least concern, *NT* near threatened, *CR* critically endangered, *DD* data deficient

A total of 73 folk binomials resulted from the interviews (Jackknite 1 Estimator calculated an expected value of 75 binomials), which corresponded to at least 23 scientific taxa, with a mean of 3.17 binomials per taxa. Some species, such as *A. superciliosus* and *Sphyrna lewini*, had four binomials each. Once the cutoff point of 15% of citation is adopted, then a total of 21 main folk species and eight synonymies were recorded. The folk species *cação-lixa* (*G. cirratum*), *cação-panã* (*Sphyrna zygaena*), and *cação-raposa* (*A. superciliosus*) were the most cited folk names, with 80%, 72%, and 67% of citations per species, respectively.

Some species, belonging to the same genera, were named by the same ethnospecific terms. Specifically, *Rhizoprionodon lalandii* and *R. porosus* were both recognized as *cação rabo-seco*, whereas *Sphyrna lewini*, *S. mokarran*, and *S. zygaena*, as *cação-panã*. The species *Negaprion brevirostris*, *Carcharhinus falciformis*, and *C. obscurus* were less recognized by fishers (more than 50% of the fishers provided no identification for them), placing them among the most misidentified species (~ 90% of misidentification per species). Besides the species identified through the photographs, fishers also mentioned the occurrence of *cação viola* (Rhinobatidae), *cação espadarte* (Pristidae), and *cação espinho* (Squalidae) in the region.

The shark species classification observed here was strongly related to external morphological attributes, exhibiting ethnotaxonomic detailing down to specific level. According to the ethnobiological classification proposed by [19], the outer hierarchical category verified in this study corresponded to “fish” (living form), which is divided into “scaly fish” and “non-scaly fish” subcategories, which is then followed by the *cação* group (corresponding to the generic level) and its folk species. Shark folk species were classified by fishers according to the animal size: small-sized, within 1.5-m length, and large-sized, above 1.5 m.

**Fig. 2:**
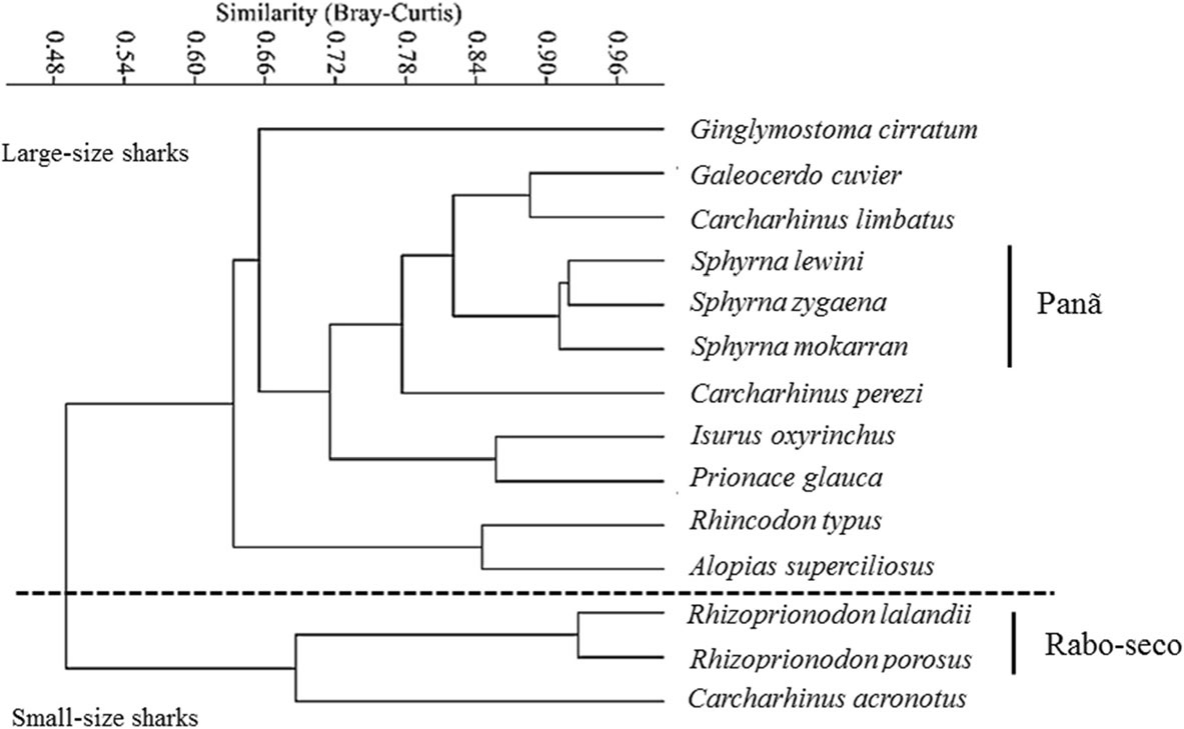
Dendrogram of the main shark species identified by small-scale fishers from the state of Rio Grande do Norte, northeastern of Brazil (30 respondents), based on external morphological attributes (size, shape, and color)

The UPGMA analysis supports the idea that fishers classify sharks mainly accordingly to morphological criteria. The categorization by size groups (small/large size) was confirmed (Fig. 2). Within these groups, there are subgroupings of high morphological similarity formed by distinct species: *Rhizoprionodon lalandii* and *R. porosus*, both named locally as *rabo-seco*, and *Sphyrna lewini*, *S. mokarran*, and *S. zygaena* known as *cação-panã*. The grouping also resulted in putting together species relatively similar, such as *Galeocerdo cuvier* and *Carcharhinus limbautis*, although these were identified under different folk names.

### Fishing and commercialization of the main shark species

Most fishers (97.72%) reported catching sharks captures within the last 12 months, mainly as bycatch from commercial fisheries aimed at snappers, flying fish and tuna, mostly. By citation rank, shark catches were significantly associated to handline (60.93%), gillnet (17.38%), and longline (13.86%) (*χ*^2^ = 265.21; d.f. = 8; *p* = 1.0228E−52). Based on fisher’s information, most sharks species caught in the studied region were as follows: *Sphyrna* spp*.* (172 citations), *Isurus oxyrinchus* (74 citations), *Rhizoprionodon* spp*.* (63 citations), *Prionace glauca* (62 citations), and *Carcharhinus acronotus* (36 citations), corresponding to over 85% of the total citations. Most of these species are currently classified as vulnerable, endangered, and near threatened in Brazil and accordingly to the IUCN (International Union for Conservation of Nature) Red List (Table 1).

**Fig. 3:**
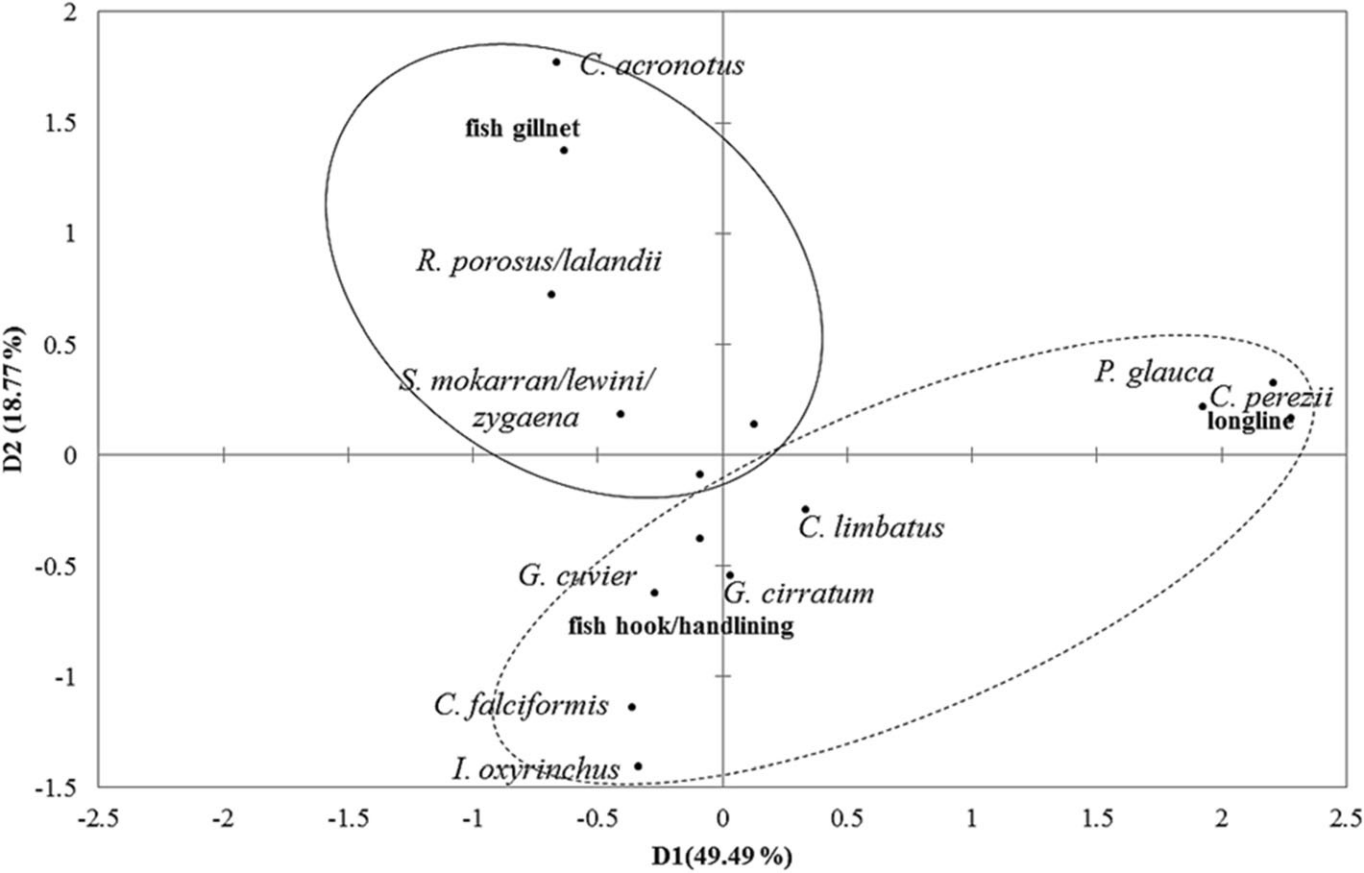
Multiple correspondence analyses for fishing period, species, and fishing gear associated to shark catches in the state of Rio Grande do Norte. Ellipses in solid line: dry season; dashed line: rainy season (eigenvalue and adjusted inertia (%): D1 = 0.570; 49.49%; D2 = 0.479; 18.77%)

The multiple correspondence analyses (MCA) performed for fishing gear, fishing season, and shark species occurrence cited by fishers indicated an association (D1 + D2 = 68.27%) among the catch of *C. acronotus*, *R. porosus*, *R. lalandii*, *S. lewini*, *S. mokarran*, and *S. zygaena* with gillnets during the dry season (from September to February). In the rainy season (from March to August), the species *Prionace glauca* and *C. perezi* were associated to longline, and *C. limbatus* and *Galeocerdo cuvier* to handline (Fig. 3).

The fisheries statistics provided by the municipalities for 2015 and 2016 registered less than 5% of sharks from an overall fishery production of 5.3 t/year. Based on the interview, 13% of sharks are landed without their head and fins, whereas the remaining sharks are roughly processed into fillets and distributed to local and nearby markets by middlemen. Small sharks (up to 3 kg) are frequently consumed by fishers and their families whereas larger animals are commercialized, generating an extra income (58.17% citations).

The market price for shark meat at the fishing communities is lower than other exploited fish groups, such as snappers. In general, snappers are high-quality fish, locally costing USD (PPP) 14.97 per kilo. In contrast, shark species such as *Prionace glauca*, *Rhizoprionodon porosus*, and *R. lalandii* hardly cost more than USD (PPP) 6.5 per kilo. According to the respondents, shark low market price is due to local dislike for the meat taste and texture.

Nevertheless, shark fin market constitutes an alternative trade. Fins from large-sized animals may be sold for over USD (PPP) 42.00 per kilo. For this reason, 93% of landed sharks get their fins removed to be sold apart. The shark fins, according to the interviewees, are directed to Asian markets, mainly Japanese and Chinese. Even though sharks are currently not classified as a target fishing group by small-scale fisheries in the region, fishers recognize their catch as an important income source.

## Discussion

Small-scale fisheries are among the traditional activities developed on coastal communities that often enable those practicing it to recognize aquatic environmental patterns and process them as practical knowledge through improvements across generations. Moreover, for those communities, fishing is an important subsistence and income source [33, 34]. In the studied region, fishing is conducted by traditional fishers whose fishing technology is mainly composed by simple vessels and artisanal fishing gear, which allow low fishing autonomy restricted to a few days at sea within inshore zones. Despite the relative simplicity, small-scale fisheries respond historically for the majority of the fish landings in the Northeastern region [35].

The species *R. porosus*, used as a positive control, has been identified by 53% of the fishers, more than the 15% minimum stipulated for a satisfactory response in this study. The minimum 15% of identification is expected to species that are commonly sighted or caught, which is the case for *R. porosus* in the studied region [23, 30]. The negative control species, *N. brevirostris*, was identified by 13% of the interviewees, one of the lowest scores registered, also expected for a species not commonly occurring in the areas visited by the small-scale fishers approached [32, 36]. Together, the level of identification of these two species supports the reliability of the information provided by the interviewed fishers.

Fishers identified sharks by generic and binomials terminologies, which reflect the relevance and involvement of the studies’ traditional communities with these fishing resources. Human populations presenting close relationships to living beings tend to classify them into more specific categories as consequence of detailed knowledge they have developed through time [19]. In contrast, societies that show more remote interactions with nature tend to exhibit lower detailing of the explored living beings, maybe due to lower utilitarian meaningfulness of them as resources in those social contexts [34–36].

Across the globe, human populations have intensively attributed names and, therefore, different meanings for living beings, mostly under utilitarian perspectives [37]. A pioneer study on ethnoichthyology was conducted in the Cha-cha fishing community from St. Thomas, Virgin Islands. There, fish identification was based on the animal morphology and mainly on their ecological behavior. Sharks, unlike bony fish, had low value for food, and therefore, different species were commonly named by general folk names [38].

Shark species identified by fishers in this study were classified down to specific level. In total, 73 binomials were recorded, all of them generated from the junction of the polytypic generic name *cação* and an ethnospecific modifier term. Alongside the Northeastern coast of Brazil, studies concerning fishers ecological knowledge have exhibited high diversity of binomials for sharks, identifying the sharks by salient characteristics, mostly morphological features [12, 39, 40]. The diversity of binomials reflects the close involvement those traditional fishing communities have developed towards the occurrence of coastal sharks in the region.

Fish folk nomenclature is subject to geographic variations as fishery resources may develop a variety of meanings under different social contexts [18, 35]. In this study, different species belonging to the same genera were commonly identified as a single folk species (e.g., *Rhizoprionodon lalandii* and *R. porosus* were both named *cação rabo-seco*). The fact that fishers may find it difficult to identify shark species belonging to the same genus has been suggested before for Brazil [41, 42], and it is probably related to the high morphological similarity between close species. The difficulty in identifying sharks to species level is also observed in the official fisheries statistics, where sometimes even species not so closely related are grouped together.

The lack of basic information on aspects, such as species composition at landings, limits the possibilities for the proper management of sharks. Therefore, any management suggestion, based on information from landings, should be seen with caution, especially if it proposes long-term measures or measures of large geographic coverage [3]. The difficulty in identifying landed shark species is being minimized by increasingly lower costs of molecular genetic techniques (for example, DNA barcoding) in global fisheries [43–45], therefore becoming an alternative to aid the identification and conservation of sharks in developing countries [46]. Affordable molecular tools may help refine data on shark catches, especially when a first ethnobiological approach is not conclusive.

The most captured and landed sharks in the studied region comprise threatened and vulnerable species at the national and global levels. Their captures, mostly as bycatch from high value commercial fisheries, represent less than 5% of the landings. This figure is common to roughly 90% of the sharks and rays captured across the world [1, 22]. However, comprising a small part of the landings, instead of representing a positive perspective, is still worrisome information. Given sharks’ biological features and their life history (slow growth, low fecundity, longevity, slow maturation), even small-scale fisheries may negatively impact local stocks. For instance, the decline in stocks of *Shyrna lewini* and other pelagic sharks in Brazil in the last decades is associated to intensive large-scale and also coastal fisheries [47]. Moreover, it is also important to consider that shark catches by small and mid-scale fisheries might have been underestimated or not even taken into account on the national or local records, as a side effect of insufficient investment and efforts from the government in fisheries management [3].

On the other hand, shark fisheries were indicated as an extra income for the small-scale fishing communities studied. Subsistence activities, as in most traditional fisheries, are often directed to make use of species of no or low commercial demand [35]. In general, sharks are consumed and commercialized at low prices along the studied coast. Body parts, such as head and fins, are frequently removed and sold apart, leading consumers to have access to mischaracterized sharks and helping explain why these fish are simply sold as “cação” in Brazil [48]. The specimen integrity comprises the basis to conventional taxonomic identification for sharks [27]; thus, mischaracterization on landings makes it difficult to identify the species, while it also facilitates the commerce of endangered species due to the lack of consumers’ awareness [2, 49].

The fact that fishers easily identified a considerable number of endangered shark species may be suggesting that they are still regularly caught (or were in a close past). Collaborative projects between traditional knowledge and scientific institutions have contributed to solving management problems in marine environments, reducing conflicts between parts, while also seeking for sustainable approaches [50]. Fishers’ expertise on identifying and classifying shark species locally, especially endangered and vulnerable ones, may be one such affordable alternative to integrate folk knowledge into government actions towards conservation plans.

The fishing gears mentioned to catch more sharks in the studied small-scale fisheries were handline and gillnet (75% of total reported catch). Such generic gears are commonly used by commercial fisheries on the continental shelf of the Brazilian Northeastern coast [51], a region where fisheries are usually done with low technology and based on multispecific gear, leading to unspecific catches [35]. Such gear mainly catch sharks during the rainy season, which may be related to increased nutrients and, consequently, higher biological resources availability in the coastal zones. More productive shallower waters make them attractive to a range of species in tropical countries [52]. Moreover, some inshore spots have been pointed out as nursery areas for at least four shark species in the studied region [23], which explains the relative diversity of shark landings on its coast.

## Conclusions

The folk nomenclature recorded in this study showed how sharks are identified, named, and classified by fishers in the northeastern of Brazil down to specific level, reflecting the tight relationship and significance of these resources to local fishing communities and nearby regions.

Folk shark identification may contribute to conservation and management plans to most recorded species. However, the identification of species morphologically similar, such as some within the same genus (e.g., *Rhizoprionodon* and *Sphyrna)* may be limited and should be taken with caution. In general, sharks are caught as bycatch of target commercial species, mostly with handline and longline gear mainly during the rainy season. Captured sharks, after finning, are directed to either subsistence consumption or local market, as an extra income for fishers. It is evident the involvement and knowledge of the fishing communities regarding sharks occurrence and, therefore, integrating their traditional expertise may be a differential when developing conservation and management plans for sharks as fishing resources.

## Additional files

Additional File 1: General form developed for interviewing small-scale fishers from the northeastern coast of Brazil. (DOCX 91 kb)

Additional File 2: Specific interview form developed for sharks identification based on morphological and ecological features previously mentioned by small-scale fishers from northeastern coast of Brazil. (DOCX 340 kb)
