# Supplementary figures and images for: Ethnotaxonomy of sharks from tropical waters of Brazil
Source: J Ethnobiol Ethnomed. 2018 Nov 21;14:71. doi: 10.1186/s13002-018-0273-0 (PMC6249882; doi:10.1186/s13002-018-0273-0)

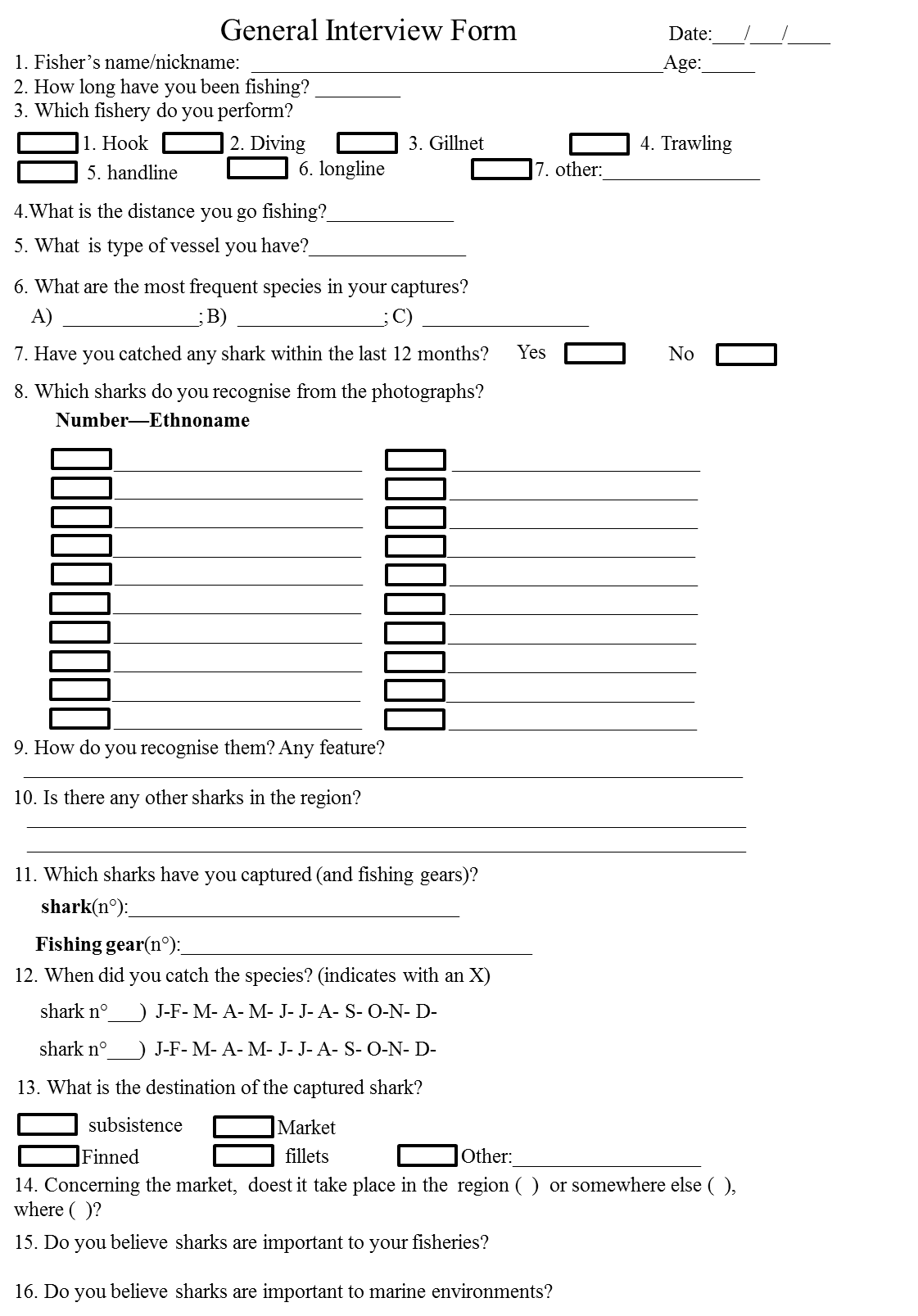

Supplement: Supplementary file 1 — General form developed for interviewing small-scale fishers from the northeastern coast of Brazil. (DOCX 91 kb) [file 13002_2018_273_MOESM1_ESM.docx]

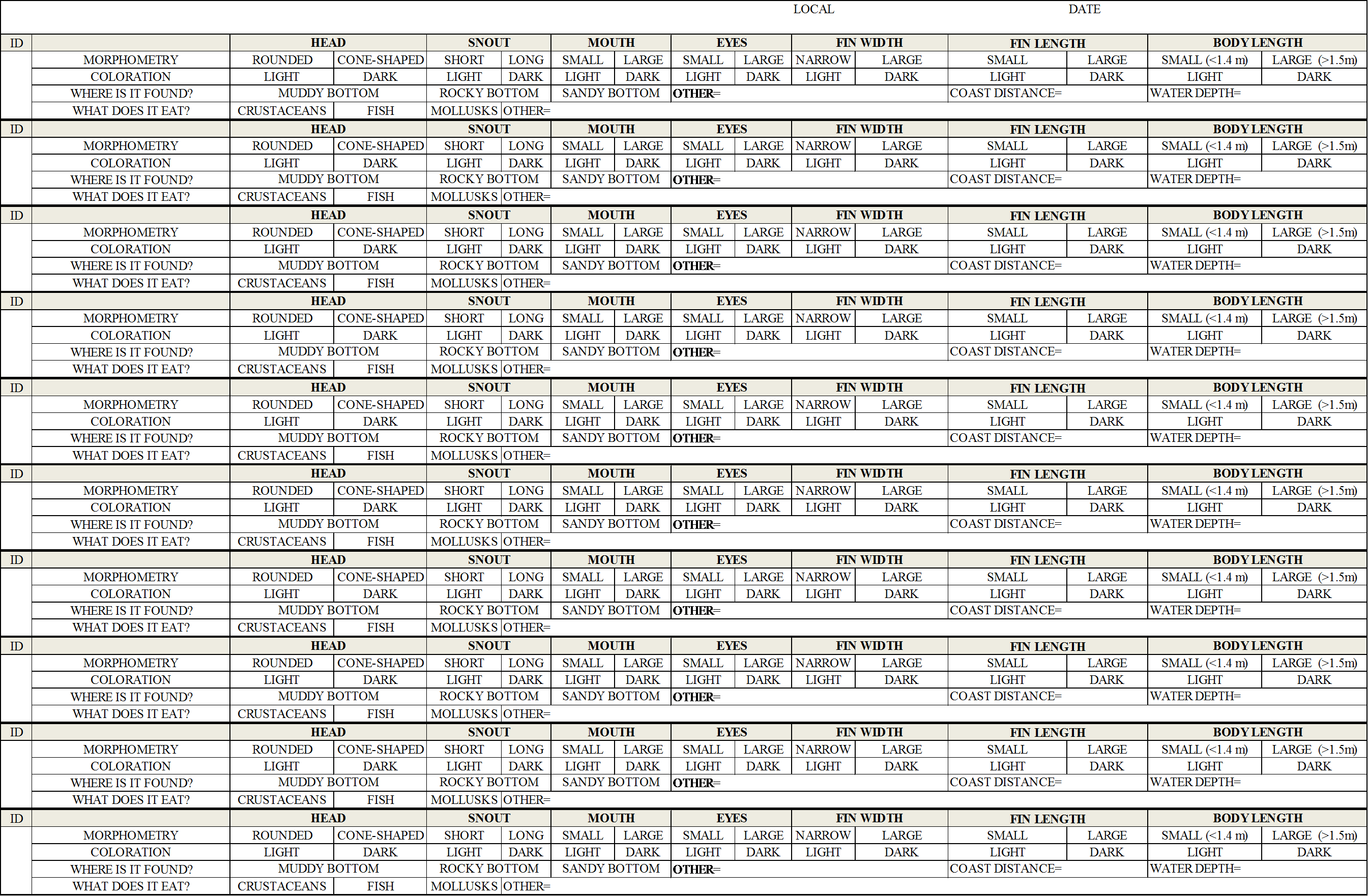

Supplement: Supplementary file 2 — Specific interview form developed for sharks identification based on morphological and ecological features previously mentioned by small-scale fishers from northeastern coast of Brazil. (DOCX 340 kb) [file 13002_2018_273_MOESM2_ESM.docx]
